# Novel molecular transport medium used in combination with Xpert MTB/RIF ultra provides rapid detection of *Mycobacterium bovis* in African buffaloes

**DOI:** 10.1038/s41598-021-86682-5

**Published:** 2021-03-29

**Authors:** Charlene Clarke, Katrin Smith, Samantha J. Goldswain, Christopher Helm, David V. Cooper, Tanya J. Kerr, Léanie Kleynhans, Paul D. van Helden, Robin M. Warren, Michele A. Miller, Wynand J. Goosen

**Affiliations:** 1 DSI‑NRF Centre of Excellence for Biomedical Tuberculosis Research, South African Medical Research Council Centre for Tuberculosis Research, Division of Molecular Biology and Human Genetics, Faculty of Medicine and Health Sciences, Stellenbosch University, PO Box 241, Cape Town, 8000 South Africa; 2 Longhorn Vaccines and Diagnostics, San Antonio, TX USA; 3 Ezemvelo KwaZulu-Natal Wildlife, PO Box 25, Mtubatuba, 3935 South Africa

**Keywords:** Molecular biology, Tuberculosis, Translational research

## Abstract

*Mycobacterium bovis* is the causative agent of bovine tuberculosis (bTB) in wildlife. Confirmation of *M. bovis* infection relies on mycobacterial culture, which is time-consuming. Collection and transportation of infectious material also pose a human health risk. PrimeStore Molecular Transport Medium (MTM) has been shown to effectively inactivate infectious organisms, making it a safe method for handling infectious samples. This study investigated an in-field sampling technique for rapid, safe detection of *M. bovis* in buffalo tissues. Potentially infected tissues from bTB test-positive buffaloes were swabbed at post-mortem examination and stored in PrimeStore MTM at ambient temperature until Xpert MTB/RIF Ultra testing was performed. Additionally, tissue samples were frozen and transported before homogenisation for culture and Ultra testing. Oral swabs were collected from *M. bovis-*unexposed buffaloes as a negative control cohort. *Mycobacterium tuberculosis* complex (MTBC) DNA was detected by Ultra in 13/16 tissue swabs and 9/16 matched tissue homogenates from culture-confirmed *M. bovis-*positive buffalo tissues. MTBC DNA was not detected in swabs from *M. bovis-*unexposed animals, showing the potentially high specificity of Ultra with PrimeStore swabs. PrimeStore MTM sample processing, in combination with the Ultra assay, has the potential to provide a safe, rapid post-mortem screening test for *M. bovis* in buffaloes.

## Introduction

Bovine tuberculosis (bTB) is an important zoonosis caused by *Mycobacterium bovis* (*M. bovis*) and is responsible for severe economic losses and disruption of conservation programs when livestock and wildlife are affected^1,2^. African buffaloes (*Syncerus caffer*) are important wildlife maintenance hosts of *M. bovis* and serve as a source of infection for other susceptible mammals sharing the same habitat^2^. This highlights the potential disease risk and importance of its control^1,2^. Diagnosis of *M. bovis* infection in African buffaloes typically relies on the measurement of cell-mediated immune (CMI) responses to *M. bovis* antigens^3^. Buffalo TB tests include in vitro interferon-gamma (IFN-γ) release assays (IGRAs) and IFN-γ inducible protein 10 (IP-10) release assays (IPRA) that use a modified QuantiFERON-TB Gold Plus (QFT) In-tube antigen stimulation platform, and the in vivo single comparative intradermal tuberculin test (SCITT)^4,5^. However, confirmation of *M. bovis* infection usually requires mycobacterial culture and genetic speciation of mycobacterial isolates cultured from tissue specimens collected during post-mortem examination. Conventional culture methods, however, have suboptimal sensitivity and are time-consuming, taking up to 56 days before results are available^6^. In addition, post-mortem examination of potentially *M. bovis*-infected buffaloes, and the collection, transport and storage of tissue specimens are considered a potential human health risk^7,8^. Infectious pathogens, other than *M. bovis,* may also be present during sample collection and transport, such as Foot-and-Mouth Disease (FMD) virus, which may lead to sample movement restrictions to laboratories due to the risk of spreading the disease. Movement restrictions placed on buffalo samples from locations with reportable diseases to laboratories may result in inadequate bTB surveillance in these areas^9^.

PrimeStore Molecular Transport Medium (MTM), used in combination with PrimeStore swabs, has been shown to effectively inactivate infectious organisms, while stabilising the nucleic acids, through lysis of the cell membranes, destruction of proteins and enzymes, and inactivation of nucleases^10^. PrimeStore MTM therefore provides a safer option for collection and transportation of samples that potentially pose a risk to human or animal health. In addition, this method may facilitate sample transport from areas that may be restricted due to the presence of reportable diseases (such as FMD), since it inactivates all pathogens.

The rapid Xpert MTB/RIF Ultra (Ultra) assay is an automated cartridge-based semi-quantitative nested real-time PCR assay that detects *Mycobacterium tuberculosis* complex (MTBC) DNA and rifampicin resistance in clinical specimens^11^. The assay targets the multicopy sequences, IS*6110* and IS*1081*, which greatly improves the detection of DNA in paucibacillary samples. IS*1081* and IS*6110* are exclusively found in MTBC members, which makes it ideal for samples collected from animals that may be infected with members of the MTBC^12,13^. Although the Ultra is typically used for detection of MTBC DNA in human samples, it has been shown to accurately detect *M. bovis* in wildlife specimens from white rhinoceros (*Ceratotherium simum*) and elephants (*Loxodonta africana*)^13^, and several other wildlife species^14,15^.

Therefore, the aim of this study was to investigate the combined use of the Ultra assay with PrimeStore MTM swab samples as a new screening technique for the detection of MTBC DNA in buffalo tissue specimens, which could provide a rapid and safer method for sample collection and transport, or in-field testing.

## Results

Sixteen tissue samples collected from 13 buffaloes were confirmed to be *M. bovis* positive by mycobacterial culture and PCR-based speciation (Table 1). Each of these 16 culture-confirmed *M. bovis* positive tissue samples had a matching PrimeStore swab sample collected during post-mortem examination and stabilised in PrimeStore MTM, and a pre-culture tissue homogenate aliquot. Both the swab and tissue homogenate were tested with the Ultra assay prior to mycobacterial culture. The Ultra assay detected MTBC DNA in 13 of 16 (81.3%) tissue swab samples, while MTBC DNA was detected directly in 9 of 16 (56.3%) tissue homogenates from culture-confirmed *M. bovis-*positive specimens. The difference between the proportion of positive Ultra assay results from tissue homogenates compared to tissue swabs was not significant using the McNemar’s test (*p* > 0.05). The agreement between these tests were regarded as “fair” on the kappa agreement scale, with κ = 0.259 (95% confidence interval (CI) − 0.2 to 0.72 and standard error (SE) of 0.23).

**Table 1:** Ultra assay results from PrimeStore MTM tissue swab samples and tissue homogenates from culture-confirmed *M. bovis* positive African buffaloes (n = 13). Positive results are shown in bold.

Buffalo number	Culture-confirmed *M. bovis* positive tissue samples	Ultra result for PrimeStore MTM tissue swabs	Ultra result for tissue homogenates
**A107**	Left retropharyngeal LN	**MTB trace detected**	**MTB detected low**
**A113**	Tonsils	**MTB trace detected**	MTB not detected
**A20**	Tonsils	**MTB detected very low**	MTB not detected
**A98**	Retropharyngeal LN	**MTB trace detected**	**MTB trace detected**
**A98**	Tonsils	MTB not detected	MTB not detected
**B15**	Lung lesion	**MTB detected low**	**MTB detected very low**
**B19**	Retropharyngeal LN	**MTB trace detected**	**MTB detected very low**
**B30**	Lung lesion	MTB not detected	MTB not detected
**B30**	Mediastinal LN	**MTB trace detected**	MTB not detected
**B48**	Right retropharyngeal LN lesion	**MTB detected very low**	**MTB detected low**
**B64**	Abdominal serosa	**MTB detected very low**	**MTB detected low**
**B65**	L/R tracheobronchial LN	**MTB detected very low**	**MTB detected very low**
**B8**	L/R tracheobronchial LN	**MTB detected very low**	**MTB detected low**
**C22**	Scalpel	MTB not detected	**MTB detected low**
**C28**	Retropharyngeal LN	**MTB trace detected**	MTB not detected
**C28**	Tonsil lesion	**MTB detected very low**	**MTB detected very low**

Ultra—Xpert MTB/RIF Ultra; LN—Lymph node; L/R—Left and right*;* MTM—molecular transport medium.

Additionally, Ultra assay did not detect MTBC DNA in any oral swabs collected from the 12 randomly selected *M. bovis* unexposed buffaloes from historically bTB free buffalo herds (data not shown).

## Discussion

The findings from this study show that the Ultra assay combined with tissue swabs that were inactivated in PrimeStore MTM can accurately identify culture-confirmed *M. bovis* positive tissues from African buffaloes. The swabs taken directly from dissected tissues and later tested on the Ultra assay performed similar to the Ultra on tissue homogenates prepared prior to culture. Recent studies have shown that Ultra provides accurate, rapid diagnosis of MTBC infection in livestock and wildlife when performed on tissue homogenates^13–15^. However, the combined use of PrimeStore MTM and tissue swabs with the Ultra assay have the added advantage of providing more rapid test results, since it can directly be used in the Ultra assay without the extra steps of tissue processing; results may even be acquired in-field if portable Ultra equipment is available (such as Cepheid’s GeneXpert Edge system), providing same day results. Typically, tissue samples are kept frozen at − 20 °C and transported to a biosafety level 3 (BSL-3) facility before being homogenised to perform the Ultra test. PrimeStore MTM incubated swabs do not require refrigeration and since PrimeStore MTM inactivates pathogens, samples could even be transported through standard postal services without biosafety concerns. Furthermore, a BSL-3 facility is not required for sample processing of swabs in PrimeStore MTM and samples can be handled safely without the risk of infection to other animals and humans.

There was a greater number of positive Ultra results using swabs than tissue homogenates. However, the difference was not significant and there was only fair agreement between the two methods. There could be various reasons for the discordant Ultra results between tissue swabs and matching tissue homogenate samples. PrimeStore MTM has been shown to inactivate nucleases in samples. Since the tissue homogenate samples were not incubated in stabilising media (PrimeStore MTM), there may have been degradation of DNA in some samples during freeze–thaw cycles, which could result in a negative Ultra result, especially if the samples were paucibacillary. Tissue swabs, however, did not undergo a freeze–thaw cycle prior to testing with Ultra. Also, since Ultra results are influenced by bacillary load^16^, swab samples from tissues with visible lesions are more targeted than taking an aliquot of the tissue homogenate for Ultra testing, which may result in some dilution of mycobacteria in a larger homogenate volume than the swabs.

Studies performed with human specimens have shown that Ultra results provide a measure of the bacillary load in a specimen, with a positive correlation between the semi-quantitative Ultra results and detection of acid-fast bacilli with smear-microscopy^17^. The “MTB trace detected” and “MTB not detected” Ultra results have been shown to be correlated with no acid-fast bacilli detected, whereas Ultra results “MTB detected high” and “medium” typically correlate with positive smear-microscopy results^17^. The Ultra test results in this study included “MTB detected low”, “MTB detected very low”, “MTB trace detected” and “MTB not detected”. Since the “MTB trace detected” Ultra results from tissue swabs and homogenates were seen in culture-confirmed *M. bovis* positive specimens, it may indicate greater sensitivity of this method compared to smear microscopy. However, varying copy numbers of IS*6110* and IS*1081* between MTBC members and between strains have also been shown to influence the sensitivity and may not entirely relate to the performance of the Ultra assay^11,13^. The bacillary load in samples in which no MTBC DNA were detected (“MTB not detected”) was likely below the threshold for detection by the Ultra assay, although the homogenates from the same tissue sample had a positive *M. bovis* result by mycobacterial culture and PCR speciation. This could be due to a low but viable bacillary load that would have increased to a detectable level during the 56 days of mycobacterial culture, which could further explain the discordant results between mycobacterial culture (all *M. bovis* positive) and Ultra results from tissue homogenates. Difference in sample volume between mycobacterial culture and Ultra from tissue swabs may also contribute to discordant results between these two methods.

The oral swabs from the *M. bovis-*unexposed buffaloes were all negative on the Ultra assay. These results are consistent with the reported high specificity of the Ultra assay^11^. Interestingly, although these swabs were used as negative controls, the swabs would have had a greater likelihood of contamination than those collected post-mortem, since these animals were sampled in a dusty, natural environment, where they were likely exposed to various environmental organisms.

The study had several limitations. Firstly, there was a small sample size of culture-confirmed *M. bovis-*positive buffaloes and oral swab samples available from the *M. bovis-*unexposed buffaloes, which did not allow for direct comparison of the same sample type tested in the Ultra. Secondly, since the Ultra assay was only performed in the laboratory, we were unable to compare laboratory and in-field testing with the Ultra assay and PrimeStore sampling platform. Finally, since tissue samples for Ultra were specifically selected based on visible lesions, it is unknown how the Ultra would perform using *M. bovis* culture-positive tissue samples with no visible lesions and therefore presumed low bacillary load. Future studies should include a larger sample size with buffaloes at various stages of infection. The application of field testing should also be investigated, which would allow more rapid sample processing and the use of fresh samples. Additionally, pooling swab samples from each buffalo should be investigated as a method to decrease the number of samples tested.

The Ultra assay with samples stored in PrimeStore MTM shows promise as a rapid post-mortem screening test, which could be used to identify MTBC DNA in tissue samples and ultimately identify infected herds more rapidly than mycobacterial culture. This test can also be used to decrease the number of samples sent for mycobacterial culture by identifying those that contain mycobacterial DNA. In addition, the use of PrimeStore MTM to safely store and transport infectious samples will safeguard human and animal health as well as permit greater use of samples that may otherwise be restricted from being transported to diagnostic laboratories.

## Materials and methods

### Animals and post-mortem sample collection

**Figure 1:**
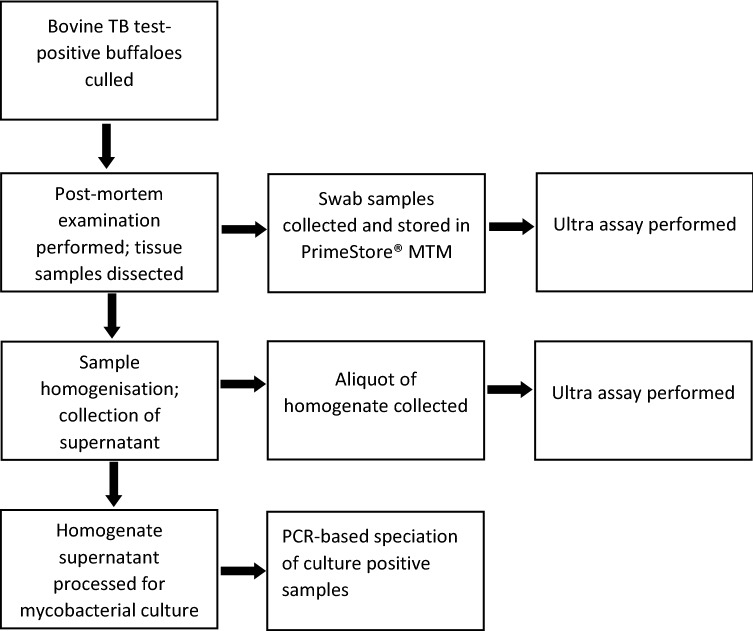
Study method flow chart for African buffaloes from the bTB-endemic game reserve, HiP*.*

The Hluhluwe-iMfolozi Park (HiP) in KwaZulu-Natal, South Africa, is a game park that is endemic for *M. bovis-*infection in the wildlife population. During the 2019 annual test-and-slaughter bTB control program, African buffaloes were captured, immobilised, sampled and tested for bTB, using the testing regime previously described^18^. Bovine TB test-positive buffaloes (n = 22) underwent post-mortem examination as previously described^18^. Various tissues with gross pathological lesions consistent with bTB^18^ were dissected and swabbed during post-mortem examination using PrimeStore swabs (Longhorn Vaccines and Diagnostics, San Antonio, Texas, USA) and stored in PrimeStore MTM (Longhorn Vaccines and Diagnostics) at ambient temperature (which may reach temperatures up to 25 °C during winter in HiP) until further processing. Tissues included retropharyngeal lymph nodes, tonsils, lung, mediastinal lymph nodes, abdominal serosa, and tracheobronchial lymph nodes. In addition, the scalpel blade used to cut tissues for a particular animal was also sampled on some occasions (Fig. 1). Samples with visible lesions were stored in separate containers and frozen at − 20 °C until processed for mycobacterial culture. Since tissue samples were not available from *M. bovis-*unexposed buffaloes, oropharyngeal swabs were collected from 12 randomly selected buffaloes that were tested for bTB in historically bTB-negative private game parks, as previously described^18^. PrimeStore swab samples were stored in PrimeStore MTM at ambient temperature until further processing. All samples were transported to Stellenbosch University for further analyses.

Ethical approval for this study was granted by Stellenbosch University Animal Care and Use Committee (ACU-2019-9081). Permission to perform animal research in terms of section 20 of the Animal Diseases Act was granted by the South African Department of Agriculture, Land Reform and Rural Development (DALRRD), formerly the Department of Agriculture, Forestry and Fisheries (DAFF), South Africa (12/11/1/7/2). All buffaloes were handled by the Ezemvelo KZN Wildlife services’ veterinarians and game capture teams according to their guidelines. ARRIVE guidelines for reporting animal research have been followed as much as possible (https://arriveguidelines.org/).

### Mycobacterial culture

All previously frozen tissue samples collected during post-mortem examination were homogenised in phosphate buffer (Becton Dickinson, Franklin Lakes, NJ, USA) with 4.8 mm stainless steel beads using a Bullet Blender 50 (Next Advance, Averill Park, NY, USA) for 15 min at maximum speed in a BSL-3 laboratory at Stellenbosch University, as previously described^3^. A 1 ml aliquot of the liquid fraction of each tissue homogenate was used for quantitative polymerase chain reaction (qPCR) analysis by the Xpert MTB/RIF Ultra (Ultra) assay (Cepheid Inc., Sunnyvale, California, United States). Tissue homogenates were then further processed for mycobacterial culture using the standard mycobacterial culture protocol with the BBL MycoPrep specimen decontamination kit (Becton Dickinson) and BACTEC MGIT 960 Mycobacterial Detection System (Becton Dickinson), as previously described^3^. Speciation by region of difference PCR was performed on all culture positive samples to confirm the presence of *M. bovis*^19^ (Fig. 1).

### Xpert MTB/RIF ultra assay

The Ultra assay (Cepheid) was performed using the oropharyngeal swabs collected from *M. bovis-*unexposed buffaloes and tissue swab samples from HiP buffaloes, according to the Ultra alternative sample assay instructions, as previously described^13^. Briefly, Xpert MTB/RIF sample lysis reagent (Cepheid) was mixed in a 1:1 ratio with each PrimeStore MTM stabilised swab sample and incubated for 10 min at room temperature (RT). Hereafter it was vortexed for 10 s before incubation at RT for 5 min followed by a 5 s vortex step. Approximately 2 ml of the prepared sample was dispensed into the sample chamber of the Ultra cartridge for automated processing. Aliquots of tissue homogenate processed for mycobacterial culture were also tested with the Ultra assay, with sample preparation as described above. The Ultra assay classifies MTBC DNA detection as high, medium, low, very low or trace, based on the quantity of MTBC DNA that was detected, and all of these classifications were considered positive in this study. When the Ultra could not detect MTBC DNA (“MTB not detected”), it was considered a negative result. Rifampicin resistance was also reported but is irrelevant in the context of this study (Fig. 1).

### Statistical analysis

Agreement between tests was calculated as Cohen’s kappa coefficient (κ) using the calculator on the GraphPad Prism Software (https://www.graphpad.com/quickcalcs/kappa2/). The McNemar’s test was used to compare Ultra results from tissue swabs and tissue homogenates using the GraphPad QuickCalcs software https://www.graphpad.com/quickcalcs/McNemar1.cfm). A *p* value < 0.05 was considered statistically significant.
